# Circadian Control of Mouse Heart Rate and Blood Pressure by the Suprachiasmatic Nuclei: Behavioral Effects Are More Significant than Direct Outputs

**DOI:** 10.1371/journal.pone.0009783

**Published:** 2010-03-22

**Authors:** W. John Sheward, Erik Naylor, Seymour Knowles-Barley, J. Douglas Armstrong, Gillian A. Brooker, Jonathan R. Seckl, Fred W. Turek, Megan C. Holmes, Phyllis C. Zee, Anthony J. Harmar

**Affiliations:** 1 Centre for Cardiovascular Science, University of Edinburgh, Edinburgh, United Kingdom; 2 Department of Neurology, Northwestern University Medical School, Chicago, Illinois, United States of America; 3 Institute for Adaptive and Neural Computation, University of Edinburgh, Edinburgh, United Kingdom; 4 Department of Neurobiology and Physiology, Northwestern University, Evanston, Illinois, United States of America; Vanderbilt University, United States of America

## Abstract

**Background:**

Diurnal variations in the incidence of events such as heart attack and stroke suggest a role for circadian rhythms in the etiology of cardiovascular disease. The aim of this study was to assess the influence of the suprachiasmatic nucleus (SCN) circadian clock on cardiovascular function.

**Methodology/Principal Findings:**

Heart rate (HR), blood pressure (BP) and locomotor activity (LA) were measured in circadian mutant (*Vipr2* ^−/−^) mice and wild type littermates, using implanted radio-telemetry devices. Sleep and wakefulness were studied in similar mice implanted with electroencephalograph (EEG) electrodes. There was less diurnal variation in the frequency and duration of bouts of rest/activity and sleep/wake in *Vipr2* ^−/−^ mice than in wild type (WT) and short “ultradian” episodes of arousal were more prominent, especially in constant conditions (DD). Activity was an important determinant of circadian variation in BP and HR in animals of both genotypes; altered timing of episodes of activity and rest (as well as sleep and wakefulness) across the day accounted for most of the difference between *Vipr2* ^−/−^ mice and WT. However, there was also a modest circadian rhythm of resting HR and BP that was independent of LA.

**Conclusions/Significance:**

If appropriate methods of analysis are used that take into account sleep and locomotor activity level, mice are a good model for understanding the contribution of circadian timing to cardiovascular function. Future studies of the influence of sleep and wakefulness on cardiovascular physiology may help to explain accumulating evidence linking disrupted sleep with cardiovascular disease in man.

## Introduction

Mammals exhibit daily rhythms in many aspects of their physiology, metabolism and behavior, such as sleep and wakefulness, the secretion of stress hormones, body temperature, heart rate and blood pressure. These rhythms are coordinated by a master circadian clock, situated in the suprachiasmatic nuclei (SCN) of the hypothalamus, which is synchronized to the external environment primarily by signals from the visual system, providing information about the 24-hr light-dark environment. The SCN may also receive timing information about the internal environment from the periphery, and in turn, delivers output circadian signals to the brain, peripheral nervous system and neuroendocrine pathways [1]. Peripheral circadian clocks, which exploit the same biochemical mechanism that drives the SCN clock, are found in most tissues and are thought to play critical roles in the local control of rhythms of physiology and biochemistry. Despite significant progress in elucidating the molecular basis of circadian oscillations, the mechanisms by which the circadian clock organizes daily rhythms of behavior, physiology and metabolism in mammals are incompletely understood. There are two mechanisms by which the SCN may impose rhythmicity: directly, by imposing circadian timing through neural or hormonal signals and indirectly, through effects on rhythms of behavior, such as feeding and the sleep-wake cycle [2].

The mechanisms underlying diurnal rhythms in blood pressure and cardiovascular function are of significant clinical interest, due to the prominent influence of time of day on the frequency of acute cardiovascular events such as myocardial infarction [3], sudden cardiac death [4] and stroke [5]. Disturbances in circadian function and sleep have been reported in mouse models of cardiometabolic disease [6], [7] and cardiovascular disease has been reported to develop in rodents carrying mutations that disrupt circadian function [8], [9]. Furthermore, disruption of normal circadian rhythmicity has been shown to reduce the lifespan of hamsters genetically susceptible to cardiomyopathic heart disease [10].

Vasoactive intestinal peptide (VIP) signaling is important for the maintenance and synchronization of circadian rhythms of clock gene expression and electrical activity in SCN neurons. In mutant mice that lack VIP [11]–[13] or the VPAC_2_ subtype of VIP receptor (*Vipr2^−/−^*) [11], [14]–[16], circadian rhythms of gene expression and electrical activity in individual SCN neurons are poorly synchronized and fewer cells exhibit detectable rhythms than in wild type. Consequently, such animals do not display robust circadian rhythms of physiology and behavior [11], [12], [15]–[19], although the ability of tissues outside the SCN to sustain circadian rhythms of clock gene expression is unimpaired [18]. *Vipr2^−/−^* mice are therefore a useful model in which to assess the role of the SCN in the control of physiology and behavior without the confounding effects of damaged neuronal connectivity, which inevitably occur in animals with SCN lesions. The aim of this study was to assess the influence of the SCN on rhythms of heart rate (HR), blood pressure (BP), sleep and wakefulness and locomotor activity by comparing the phenotypes of *Vipr2* ^−/−^ and WT mice.

## Methods

### Ethics statement

Sleep studies were conducted at Northwestern University using procedures approved in advance by the Institutional Animal Care and Use Committee. All other experimentation was conducted in accordance with the United Kingdom Animals (Scientific Procedures) Act, 1986 using procedures approved by the University of Edinburgh Ethical Review Committee.

### Radiotelemetric measurement of blood pressure and activity

Adult male mice (wild type WT or *Vipr2^−/−^* on a C57BL/6J background, 3–4 months old) were anaesthetized with ketamine (50 mgkg^−1^, i.p.) and medetomidine (0.75 mgkg^−1^, i.p.). Radiotelemetric catheters (PA-C10, Data Sciences International, St Paul MN) were inserted into the left common carotid artery with the transmitter implanted subcutaneously. Mice were housed individually at 22°C, initially with a 12∶12 light dark cycle (LD; lights on 0700 h, lights off 1900 h). After 5–7 days, mice had recovered from surgery and exhibited regular diurnal rhythms of activity. Measurements of heart rate, blood pressure and activity were recorded at 4-minute intervals for the duration of the study. The data from the telemetric device was collected using the Dataquest A.R.T system, version 4.0 (Data Sciences International, St Paul MN) by way of a RPC-1 receiver placed under the mouse cage. After collection of data in LD conditions for 8 days, mice were transferred to constant dark (DD) conditions for a further 8 days. During periods of darkness (in both LD and DD conditions) a dim red light was permanently on to facilitate animal care.

### Sleep studies

Seven C57BL/6 male mice lacking the VPAC_2_ receptor gene (*Vipr2* ^−/−^) along with six wild-type male, littermate controls (WT) were kept in a 12 hour light/12 hour dark cycle (LD 12∶12) prior to surgery. Animals were age (*Vipr2* ^−/−^: 8.7±0.6 months, wt: 9.3±0.3 months) and weight (*Vipr2* ^−/−^: 33.1±1.9 g, wt: 33.7±1.3 g) matched as closely as possible. Food and water were available *ad libitum* throughout the experiment.

### Recording of Sleep

Under ketamine and xylazine anesthesia mice were implanted with electrodes for EEG and EMG recording. EEG recording was accomplished via four stainless steel screws (Small Parts #MX-000120-02FL, Miami, FL) positioned at the following locations: 1.5 mm anterior of bregma, 1 mm left and right of midline, 1 mm anterior of lambda and 1 mm left and right of midline. EMG activity was monitored using Iridium/Silver alloy wires (MedWire #10IR5T, Mt. Vernon, NY) inserted bilaterally into the nuchal muscles. All electrodes were attached to a pre-fabricated head mount board (Pinnacle Technology, Inc, Lawrence, KS) and secured using dental acrylic. Mice were adapted to the recording tether for one week before 48 continuous hours of EEG and EMG waveforms were collected. Mice were then placed into DD conditions within the recording chamber for a minimum of 10 days in order to establish a free-running activity rhythm. Waveforms were then recorded for 48 hours to measure sleep under DD conditions. Analysis during this period included five *Vipr2* ^−/−^ and five WT animals, because two *Vipr2* ^−/−^ mice and one WT mouse had to be excluded from recording due to deteriorating signal quality. Tau was calculated using chi-square analysis over a period of 7–10 days Sleep records within one circadian cycle were then scored and analyzed as a percentage of time with respect to each animal's individual tau. In the case of *Vipr2* ^−/−^ mice, no discernable rhythm could be calculated by either best-fit line or chi-square analysis. To calculate sleep times in *Vipr2^−/−^* mice, average tau of the wild-type mice (23.6 h) was used.

### Data analysis of sleep/wake recordings

All signals were amplified 100X at the preamplifier stage before passing through the tether and swivel. At the main amplifier stage, EEG signals were amplified an additional 100X (10,000X total) and EMG signals were amplified an additional 50X (5,000X total). Both EEG channels were subjected to high pass filtering at 0.5 Hz and low-pass filtered at 50 Hz. EMG signals were high pass filtered at 10 Hz and low-pass filtered at 200 Hz. A 60 Hz digital notch filter was applied to all signal channels. Signals were sampled at 400 Hz and digitized using a 14-bit A/D converter (Texas Instruments ADS7871). Waveforms were collected and stored on a standard desktop PC running the Sirenia hardware and collection suite (Pinnacle Technology, Inc. Lawrence, KS). Waveform data was divided into 10 sec. epochs and classified into one of three vigilance states (wake, NREM sleep or REM sleep) based on predefined criteria [20]–[22]. Post scoring processing was accomplished using the Sirenia analysis package (Pinnacle Technology, Inc. Lawrence, KS).

### Statistical analysis

Data are expressed as mean ± SEM. The significance of the difference between means was tested by appropriate t-tests or a non-parametric Mann-Whitney U test. For statistical analysis of changes in sleep parameters over time repeated measures ANOVA with Tukey's post- hoc tests was used.

## Results

### Effects of the *Vipr2* mutation on locomotor activity

**Figure 1:**
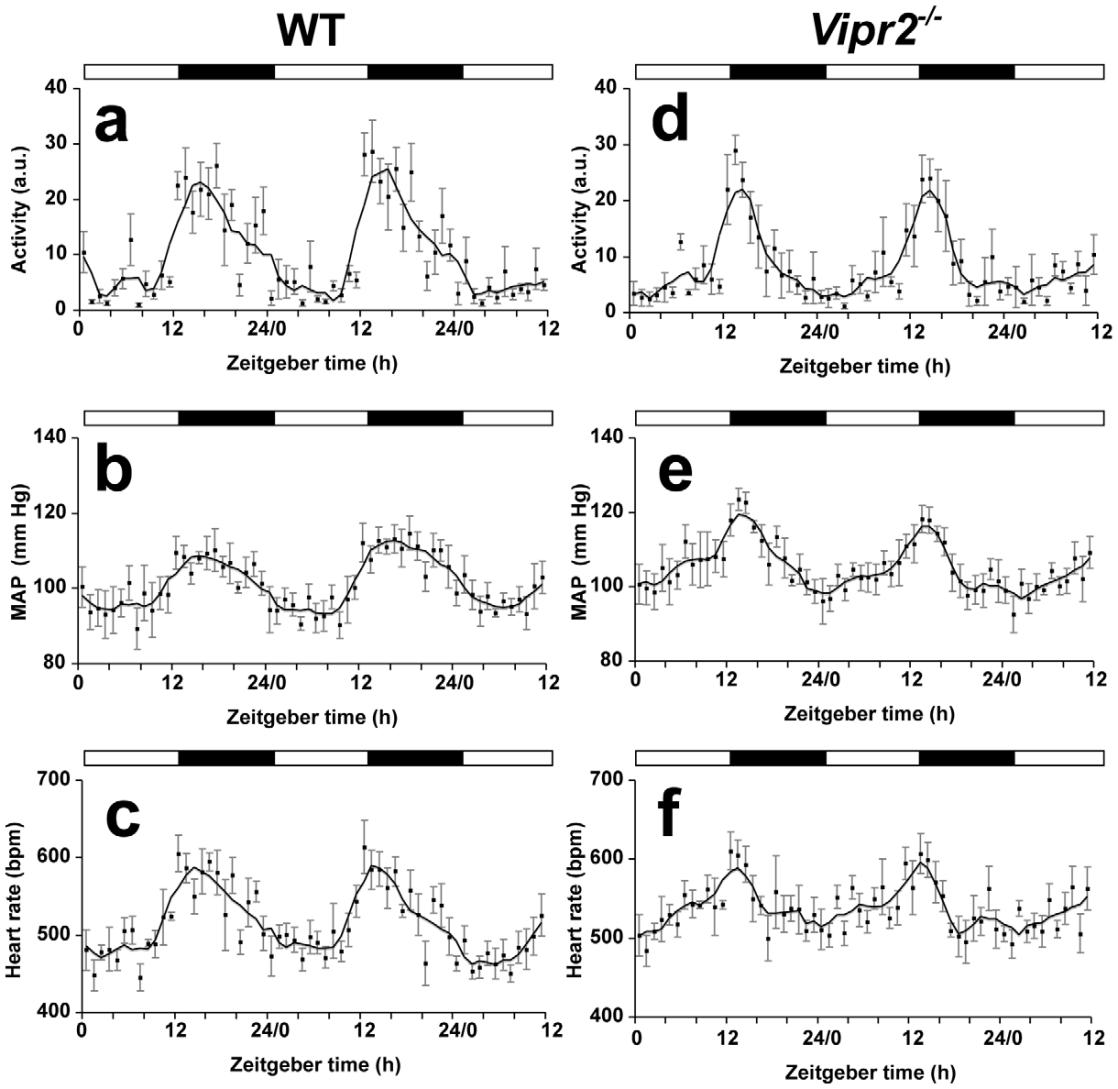
Mean activity, blood pressure and heart rate in WT and *Vipr2^−/−^* mice in a light-dark cycle. Activity (a, d), mean arterial pressure (MAP: b, e) and heart rate (c, f) were measured over 60 h. Values plotted are hourly means (± SEM, n = 5) for groups of WT (a–c) and *Vipr2^−/−^* (d–f) mice. Solid lines represent data smoothing using the weighted average of the 9 nearest points [26]. The bars at the top of each panel indicate the dark period in black and the light period in white.

**Figure 2:**
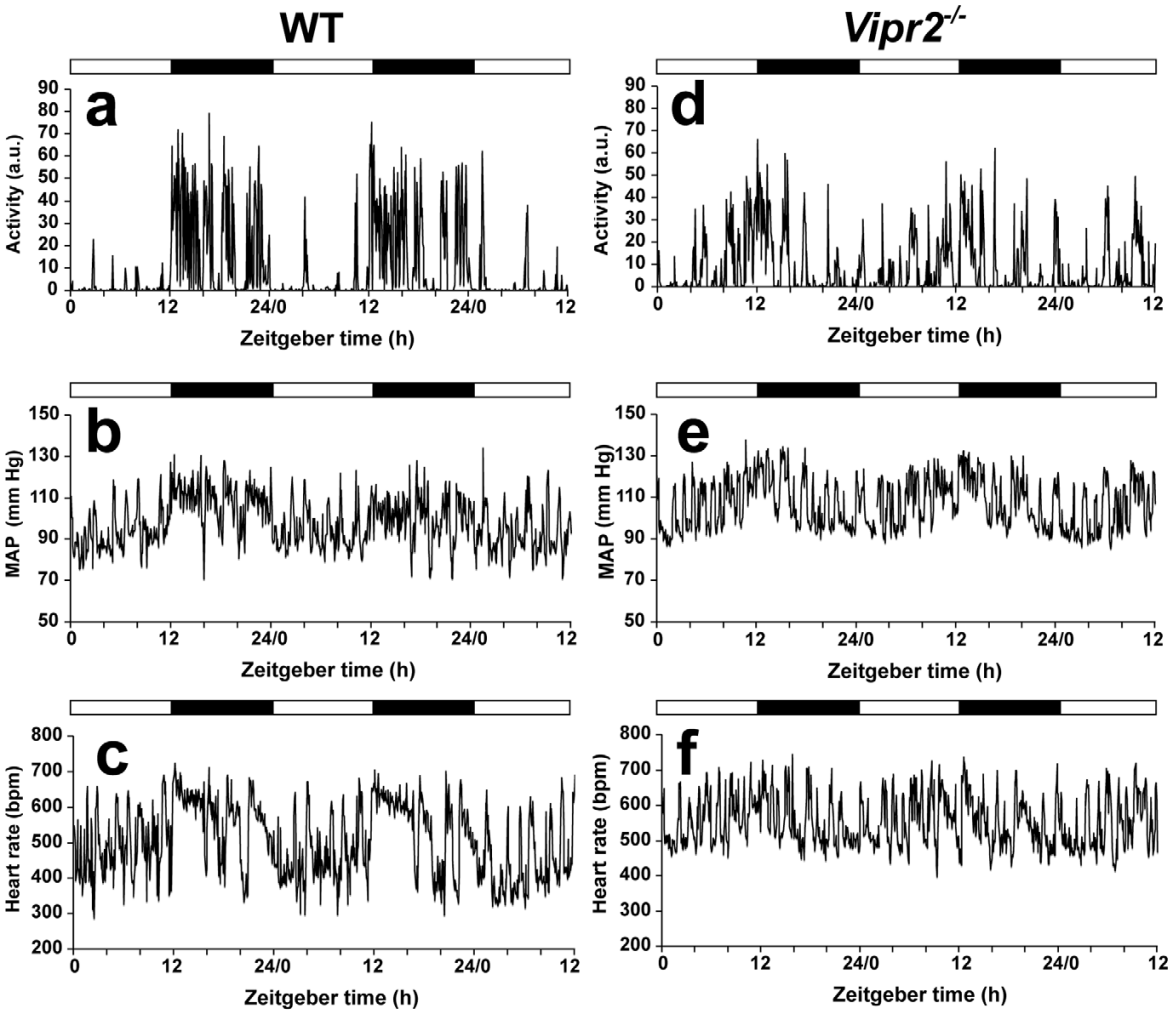
Activity, blood pressure and heart rate in individual WT and *Vipr2^−/−^* mice in a light-dark cycle. Activity (a, d), mean arterial pressure (MAP: b, e) and heart rate (c, f) were measured over 60 h in representative individual WT (a–c) and *Vipr2^−/−^* (d–f) mice. The bars at the top of each panel indicate the dark period in black and the light period in white.

**Figure 3:**
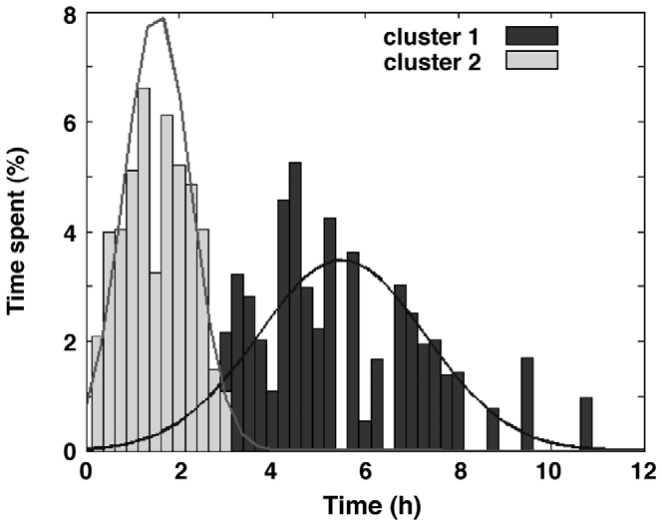
K-means clustering of the durations of periods of activity in WT mice in a LD cycle showing a bimodal distribution.

There were robust diurnal rhythms of locomotor activity in both *Vipr2* ^−/−^ and WT mice (Figure 1a, d; Figure S2), but in *Vipr2* ^−/−^ mice a greater proportion of daily activity took place in the last 8 h of the light period (25±4% vs 14±3%; *P*<0.05) and a lower proportion in the last 8 h of the dark period (29±3% vs 44±2%; *P*<0.001) than in WT. Records from individual animals (Figure 2a, d) showed that this was because *Vipr2* ^−/−^ mice exhibited less sustained activity at the beginning of the dark period and more prominent short (“ultradian”) cycles of activity and rest than WT in both light and dark periods. Serial correlation analysis of the intervals between successive activity onsets [23]–[25] indicated that the ultradian activity bouts were not generated by an oscillatory mechanism (serial correlation coefficients were 0.030±0.05 in WT and 0.128±0.083 in *Vipr2* ^−/−^ mice). To define a measure of ultradian activity that could be used to compare the behavior of mice of the two genotypes, we examined the durations of periods of activity and rest (activity signal  = 0). K-means clustering analysis (MatLab 7.0 Statistical Toolbox) revealed that the durations of periods of rest were distributed bimodally in all mice, with two components of short (“ultradian”: 1.53±0.71 h; mean ± SD) and long (5.50±1.80 h; mean ± SD) duration respectively (Figure 3). The time spent in rest periods less than 3 h in duration was thus a measure of ultradian activity, which robustly distinguished the behavior of *Vipr2* ^−/−^ mice from WT.

In WT mice, periods of rest >3 h occurred predominantly during the inactive (light) period whereas the time spent in ultradian episodes of rest and activity was similar between day and night (Table 1). *Vipr2* ^−/−^ mice spent significantly more time than WT in ultradian episodes of rest, which occurred mostly during the light period, and significantly less time in rest periods >3 h. When transferred into constant (DD) conditions, *Vipr2* ^−/−^ mice continued to display significant 24 h periodicity in their locomotor activity (Figure S2), but this was less robust than in WT and ultradian cycles of activity and rest predominated (Figure S1). In contrast, WT mice continued to display robust circadian rhythms of locomotor activity, with long periods of rest occurring predominantly during light period (Table 1 and Figure S1).

**Table 1:** Time spent in short “ultradian” and long periods of rest and sleep.

				WT		*Vipr2* ^−/−^	
				Rest	Sleep	Rest	Sleep
			day	31.5±4.7	34.0±8.6	52.8±5.1*	40.3±4.8
	Short (<3 h)		night	29.3±3.1	15.2±1.7	25.0±2.4	27.7±5.2^***^
Light-dark cycle			24 h	30.4±3.0	24.6±4.4	38.9±3.2*	34.0±3.9
(LD)			day	37.3±5.5	31.6±6.9	27.4±5.2	14.2±5.1*
	Long (>3 h)		night	12.2±4.2	0	1.4±1.4*	5.0±5.0
			24 h	24.8±3.4	15.8±3.4	14.4±2.9*	9.6±4.1
			“day”	39.1±4.5	31.9±6.4		
	Short (<3 h)		“night”	30.4±2.6	29.2±4.3		
Constant conditions			24 h	34.7±2.5	30.5±3.2	40.2±1.9	40.4±4.5
(DD)			“day”	36.9±5.6	30.0±8.1		
	Long (>3 h)		“night”	12.9±5.2	0		
			24 h	24.9±2.5	15.0±4.0	5.5±1.8**	3.7±3.7*

Percentage of time (mean ± SEM) spent in short (“ultradian”: <3 h) and long (>3 h) periods of rest (activity  = 0) and sleep (NREM + REM) in wild type (WT) and *Vipr2* ^−/−^ mice. * *P<*0.05, ** *P<*0.01, *** *P<*0.001 vs. WT.

### Effects of the *Vipr2* mutation on BP and HR

Mean values of BP, HR and locomotor activity in *Vipr2* ^−/−^ and WT mice maintained on a light-dark (LD) cycle are listed in Table S1, with periodogram analysis shown in Figure S2. Diurnal variations in HR and BP closely followed the activity profiles of animals of the respective genotypes, with *Vipr2* ^−/−^ mice displaying lower average levels of HR and BP than WT in the last 8 h of the dark period (Figure 1a–f). Examination of records from individual animals showed that BP and HR fluctuated much more dramatically between periods of activity and rest than they did across the 24 h cycle (Figure 2a–f). Thus, the timing of episodes of activity and rest across the day was clearly a major factor contributing to the diurnal rhythm of BP and HR. K-means clustering analysis showed that the durations of periods of elevated BP and HR were distributed bimodally in all mice, consistent with the bimodal distribution of periods of activity (data not shown).

**Figure 4:**
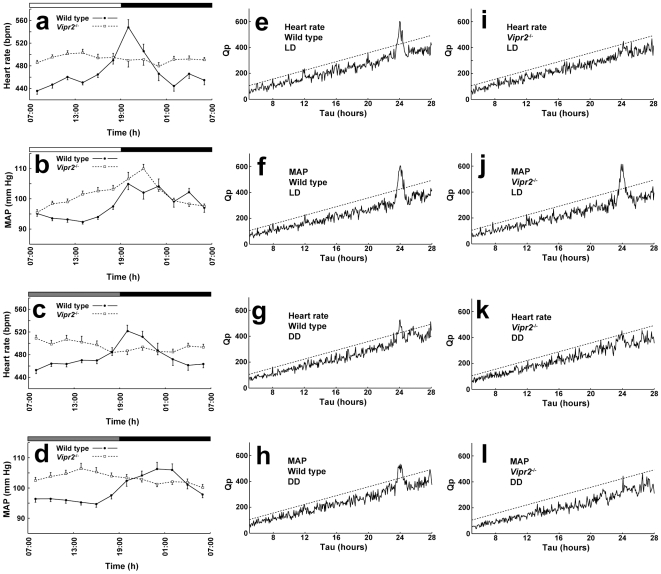
Rhythms of resting blood pressure and heart rate. Panels a – d show mean (± S.E.M., n = 5) heart rate (a, c) and MAP (b, d) during periods of inactivity, averaged over 2 h time periods across 24 h, extracted from 10 day recordings from WT (-•-) and *Vipr2* ^−/−^ (-□-) mice in a light-dark cycle (LD: a, b) and in constant conditions (DD: c, d). The bars at the top indicate the dark period in black and the light period in white (a, b) or subjective night in black and subjective day in gray (c, d). In (e–l), average Chi-square periodograms were constructed (using a formula appropriate for records containing gaps) from “binned” heart rate (e, g, i, k) and MAP (f, h, j, l) records (4 min bins) from WT (e–h) and *Vipr2* ^−/−^ (i–l) mice (n = 5 of each genotype) over 10 day periods in a light-dark cycle (LD: e, f, i, j) and in constant conditions (DD: g, h, k, l). The Qp statistic was calculated for periods between 5 and 28 h. The Qp statistic represents the degree to which each period is present in the data, after accounting for differences due to chance. Dashed lines indicate the value of Qp required to achieve statistical significance (*P*<0.01). Where significant rhythmicity was found, the estimate of τ, the circadian period, is shown.

To determine whether there were underlying circadian rhythms in BP and HR independent of the influence of activity, we analyzed data from WT and KO mice using only measurements from times when the corresponding activity signal was 0 [26], [27]. This analysis indicated that in LD, there was a significant diurnal rhythm in resting HR in WT but not in *Vipr2* ^−/−^ mice, confirmed by chi-square periodogram analysis, which peaked at around the time of the light-dark transition (Figure 4). There was also modest diurnal rhythmicity in BP in both WT and *Vipr2^−/−^* animals. In constant (DD) conditions, there were significant circadian rhythms in resting HR and BP in WT mice but not in *Vipr2^−/−^* animals (Figure 4).

We used two statistical approaches to determine the relative contributions of locomotor activity and the day/night cycle to variability in HR and BP. Using the squared correlation coefficient (***R*** ^2^) measure for explained variance [28] we found that the day/night cycle explained less of the variability in heart rate and blood pressure in WT mice than locomotor activity in all conditions (Table S2); in *Vipr2* ^−/−^ mice the contribution of the day/night cycle was very small (less than 5%). We also calculated information gain, using the Kullback-Leibler divergence, which does not assume a linear relationship [29]. This produced similar results, with the activity signal providing more information gain about HR and BP than the time of day in all conditions (data not shown).

### Effects of the *Vipr2* mutation on sleep

**Figure 5:**
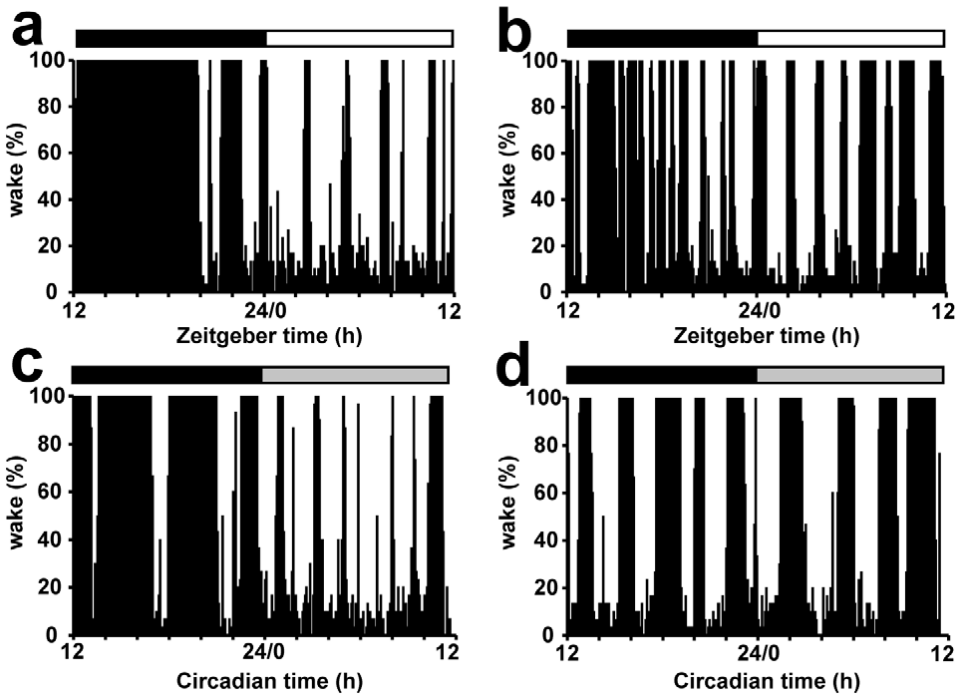
Sleep patterns of individual *Vipr2* ^−/−^ and wild-type mice under entrained (LD) and constant (DD) conditions. Representative records of wake/sleep patterns from individual WT (a, c) and *Vipr2^−/−^* (b, d) mice in LD (a, b) and DD (c, d) conditions. Values plotted are the percentage of each 5 min interval during which animals were awake; the bars at the top indicate the dark period in black and the light period in white (a, b) or subjective night in black and subjective day in gray (c, d).

**Figure 6:**
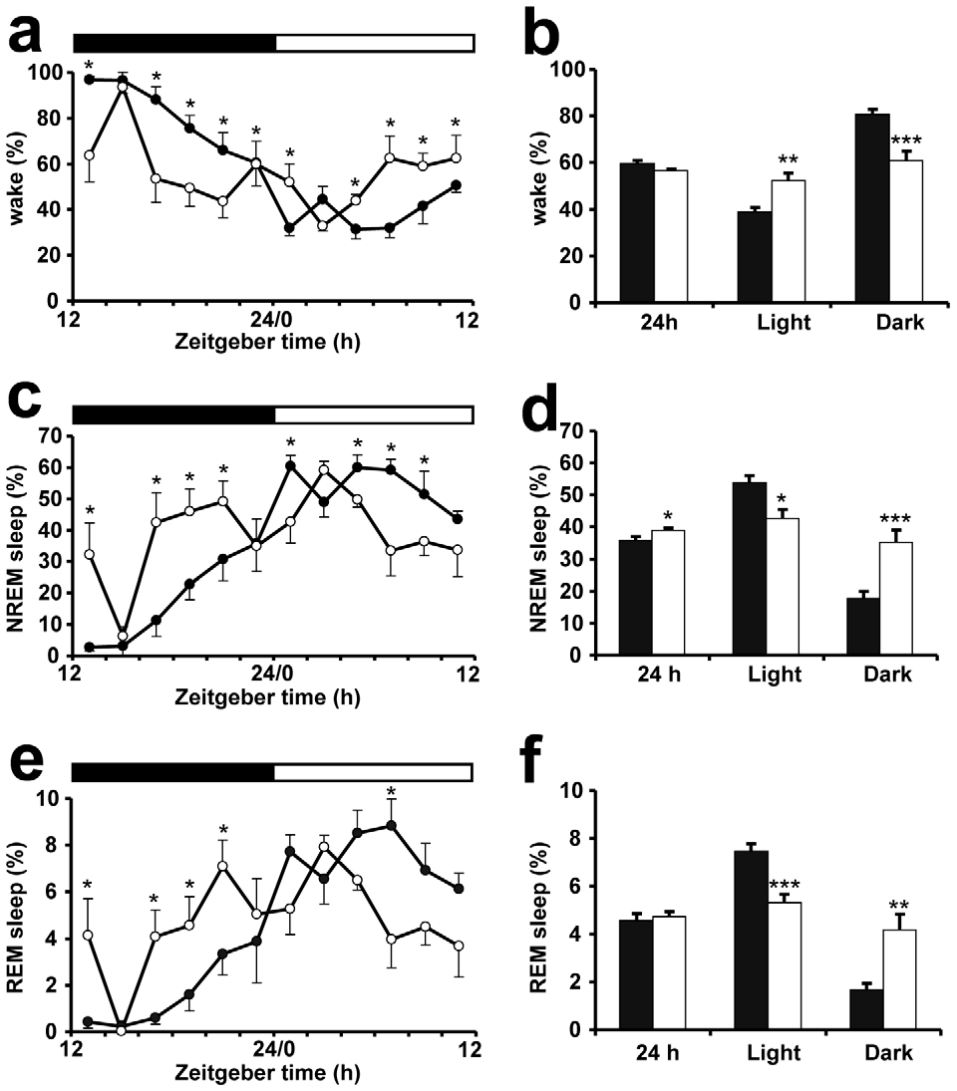
Sleep patterns of *Vipr2* ^−/−^ and wild-type mice under entrained (LD) conditions. Baseline sleep patterns for six wild-type control mice (black symbols) and seven *Vipr2* ^−/−^ (white symbols) under entrained (LD 12∶12) conditions are shown. In a, c and e, the time in each sleep stage is shown as a percentage of the recording time in 2 h intervals (mean ± SEM). The bars at the top indicate the dark period in black and the light period in white. * indicates significant differences (*P*<0.05) between mouse genotypes at that time point (RM-ANOVA df  = 1,11; Fisher's post-hoc). b, d and f show sleep times averaged over the entire 24 h recording period as well as during both the 12 h light and 12 h dark phase (mean ± SEM). Significant differences between genotypes are shown: *  =  *P*<0.05, **  =  *P*<0.005, ***  =  *P*<0.0005; *t* test for independent samples. NREM  =  non-rapid eye movement; REM  =  rapid eye movement.

Consistent with locomotor activity data, there was an altered diurnal rhythm of sleep and wakefulness in *Vipr2* ^−/−^ mice compared to WT (Figures 5,6). WT mice were predominantly awake in the dark phase and asleep during the light phase, with fewer but significantly longer wake bouts during the dark period, chiefly due to the concentrated and prolonged activity immediately following lights-off (Figures 5,6 and Table S3). In *Vipr2* ^−/−^ mice, bouts of sleep and wakefulness were more similar in number and duration between the dark and light phases. *Vipr2* ^−/−^ mice also demonstrated more stage shifts between wake, NREM and REM sleep and more arousals than WT mice. Over 24 hours, *Vipr2* ^−/−^ mice had an average of 46 minutes more NREM sleep time compared to WT mice. REM sleep, however, was similar between the two genotypes over the 24 hours.

As with LA data, the duration of periods of sleep (REM + NREM) was best fitted by a bimodal distribution, with two components of short (1.60±0.67 h; mean ± SD) and long (5.31±1.51 h; mean ± SD) duration; the time spent in periods of sleep greater and less than 3 h in duration robustly distinguished the behavior of *Vipr2* ^−/−^ mice, which spent significantly less time in periods of sleep >3 h during the light phase and more time in periods of sleep <3 h during the dark phase than WT (Table 1). In constant (DD) conditions, WT mice continued to display robust circadian rhythms of sleep and wakefulness, with periods of sleep occurring predominantly during the subjective day, whereas in *Vipr2* ^−/−^ mice short “ultradian” cycles of sleep and wake predominated at all times (Table 1, Figure 5d). During conditions of constant darkness, total wake (*Vipr2* ^−/−^ 56.2%±1.7%, wt 55.0%±0.9%; P>0.05, *t* test), NREM (*Vipr2* ^−/−^ 39.4%±1.5%, wt 40.1%±1.0%; P>0.05, *t* test), and REM (*Vipr2* ^−/−^ 4.4%±0.3%, wt 4.9%±0.2%; P>0.05, *t* test) sleep times and total NREM delta energy did not differ between *Vipr2* ^−/−^ and WT mice.

## Discussion

In this study we have shown that *Vipr2^−/−^* mice, which lack a robust SCN clock, display altered diurnal rhythms of locomotor activity, sleep and wakefulness compared to WT when entrained to a light-dark cycle. In WT mice, periods of activity and of wakefulness were largely confined to the dark period whereas *Vipr2* ^−/−^ mice exhibited less sustained activity in the dark period and more prominent short cycles of activity/rest and sleep/wake than WT in both light and dark periods. In constant conditions, ultradian cycles of activity/rest and sleep/wake predominated in *Vipr2* ^−/−^ mice, whereas WT mice displayed robust circadian rhythms of behavior. The behavioral phenotype of *Vipr2^−/−^* mice was similar to that of other mice with mutations that disable the circadian clock in all tissues of the body. Like *Vipr2^−/−^* mice, *Bmal1* null [21], *mPer1, mPer2* double mutants [30] and *Cry1*, *Cry2* double mutants [31] all display altered diurnal rhythms of activity, sleep and wakefulness in a LD cycle but become arrhythmic in constant conditions, displaying ultradian bouts of sleep and wakefulness. These ultradian rhythms appear not to be driven by an oscillatory mechanism; rather, the duration of periods of inactivity appears to be determined stochastically (randomly) [23], [32]. Studies on sleep and locomotor activity in mice [22], [33] and rats [34] with SCN lesions have used animals that were behaviorally arrhythmic in LD, suggesting that pathways required for the suppression of activity (“masking”) by light were interrupted, either through collateral damage from the lesions or because these pathways pass through the SCN. *Vipr2^−/−^* mice are therefore more informative than lesioned animals in defining the role of the SCN in the control of sleep and wakefulness and other aspects of physiology and behavior.

The importance of locomotor activity as a determinant of BP and HR in mice has been demonstrated in previous studies [26], [27], but our analysis is the first to assess the contribution of activity to circadian rhythms of cardiovascular physiology. There were robust diurnal rhythms in HR and BP in *Vipr2^−/−^* mice lacking a functional SCN clock, but these were largely due to rhythms of activity. When the contribution of locomotor activity was taken into consideration by analyzing only data from periods when animals were at rest, we found significant residual rhythms in resting HR in WT mice but not in *Vipr2^−/−^* mutants. There was also evidence for a modest influence of the circadian clock on resting BP, which was rhythmic in WT animals but not in *Vipr2^−/−^* mutants in constant conditions. A rhythm of resting BP was seen in both WT and *Vipr2^−/−^* mice in a LD cycle. We speculate that the diurnal rhythms of physiology (including activity and feeding), imposed by a LD cycle, may influence vascular tone through the entrainment of peripheral circadian clocks.

Studies of the circadian control of cardiovascular function in human volunteers, in contrast to those in rodents, can use protocols that not only remove periodic influences from the environment but also remove influences due to periodic changes in behavior (activity, food intake and sleep/wakefulness) [35]. Consistent with our findings in mice, such human studies have provided evidence for an endogenous circadian rhythm of HR, independent of the effects of activity [36]–[40]. Circadian variation of BP was absent [38], [39], perhaps because the experimental subjects were prevented from engaging in physical activity or sleep and the rate of food intake was held constant across the day. Thus, in both rodents and humans, some aspects of cardiovascular function appear to be under direct circadian control, but others may be secondary to changes in behavior.

A number of published studies [41]–[47] have reported mutations in mice that attenuate diurnal or circadian variation in BP and HR. Suprachiasmatic nucleus lesions have also been reported to abolish circadian variation in blood pressure in rats [48], [49]. However, these studies did not take into account the influence of changes in patterns of locomotor activity in such animals. In one study that did take account of activity levels when analyzing the BP rhythm [26], an apparent effect of deficiency in β_1_ and β_2_-adrenergic receptors on circadian variation in BP was no longer evident when the effects of locomotor activity were corrected for. Our findings emphasize the importance of considering the effects of activity when analyzing BP and HR data and indicate that the main influence of the circadian clock on cardiovascular physiology is mediated indirectly, through effects on arousal.

If rodents are to be useful tools for translational medicine, it is important that the findings of experimental studies in preclinical models and in humans be consistent. Activity and rest exert much more profound influences on physiology in small rodents than they do in humans. Nevertheless, our findings indicate that, with appropriate methods of analysis, mouse cardiovascular data is consistent with that obtained in human volunteers under rigorously controlled conditions. Evidence from clinical studies suggests that disturbances in sleep and sleep disorders play a role in the morbidity of cardiovascular disease [50], [51] Future studies of the influence of sleep and wakefulness on cardiovascular physiology in rodents may help to explain these links.

## Supporting Information

Figure S1 Activity (a, d), HR (b, e) and MAP (c, f) in representative individual WT (a–c) and *Vipr2^−/−^* (d–f) mice in DD conditions. The bars at the top of each panel indicate the subjective night in gray and the subjective night in black.
(3.80 MB TIF)

Figure S2 Periodogram analysis of activity, HR and MAP. Chi-square periodograms of activity (a–d), HR (e–h) and BP (i–l) from WT (a, c, e, g, i, k) and *Vipr2^−/−^* (b, d, f, h, j, l) mice (n = 5 of each genotype) over 10 day periods in a light-dark cycle (LD: a, b, e, f, i, j) and in constant conditions (DD: c, d, g, h, k, l). The Qp statistic was calculated for periods between 5 and 28 h. Dashed lines indicate the value of Qp required to achieve statistical significance (*P*<0.01).
(3.31 MB TIF)

Table S1 Summary of basic hemodynamics and activity for wild-type and *Vipr2^−/−^*mice under entrained (LD 12∶12) conditions. * *P*<0.05, ** *P*<0.005, compared with WT (unpaired t-test) †*P*<0.05, ‡*P*<0.01, §*P*<0.005, ¶*P*<0.001 compared with corresponding value in the light period (paired t-test).
(0.07 MB PDF)

Table S2 Relative contributions of locomotor activity and the day/night cycle to variability in HR and BP. Percentage (mean ± SEM) of the total variance in HR and BP explained by locomotor activity and the day/night cycle, calculated using the squared correlation coefficient (*R* ^2^) measure for explained variance.
(0.08 MB PDF)

Table S3 Number and average length of wake and sleep bouts greater than 30 sec. for seven *Vipr2^−/−^* and six wild-type mice under entrained (LD 12∶12) conditions.*P<0.05, †*P*<0.02, ‡*P*<0.01, §*P*<0.005 compared with WT.
(0.08 MB PDF)
