# Supplementary figures and images for: Circadian Control of Mouse Heart Rate and Blood Pressure by the Suprachiasmatic Nuclei: Behavioral Effects Are More Significant than Direct Outputs
Source: PLoS One. 2010 Mar 22;5(3):e9783. doi: 10.1371/journal.pone.0009783 (PMC2842429; doi:10.1371/journal.pone.0009783)

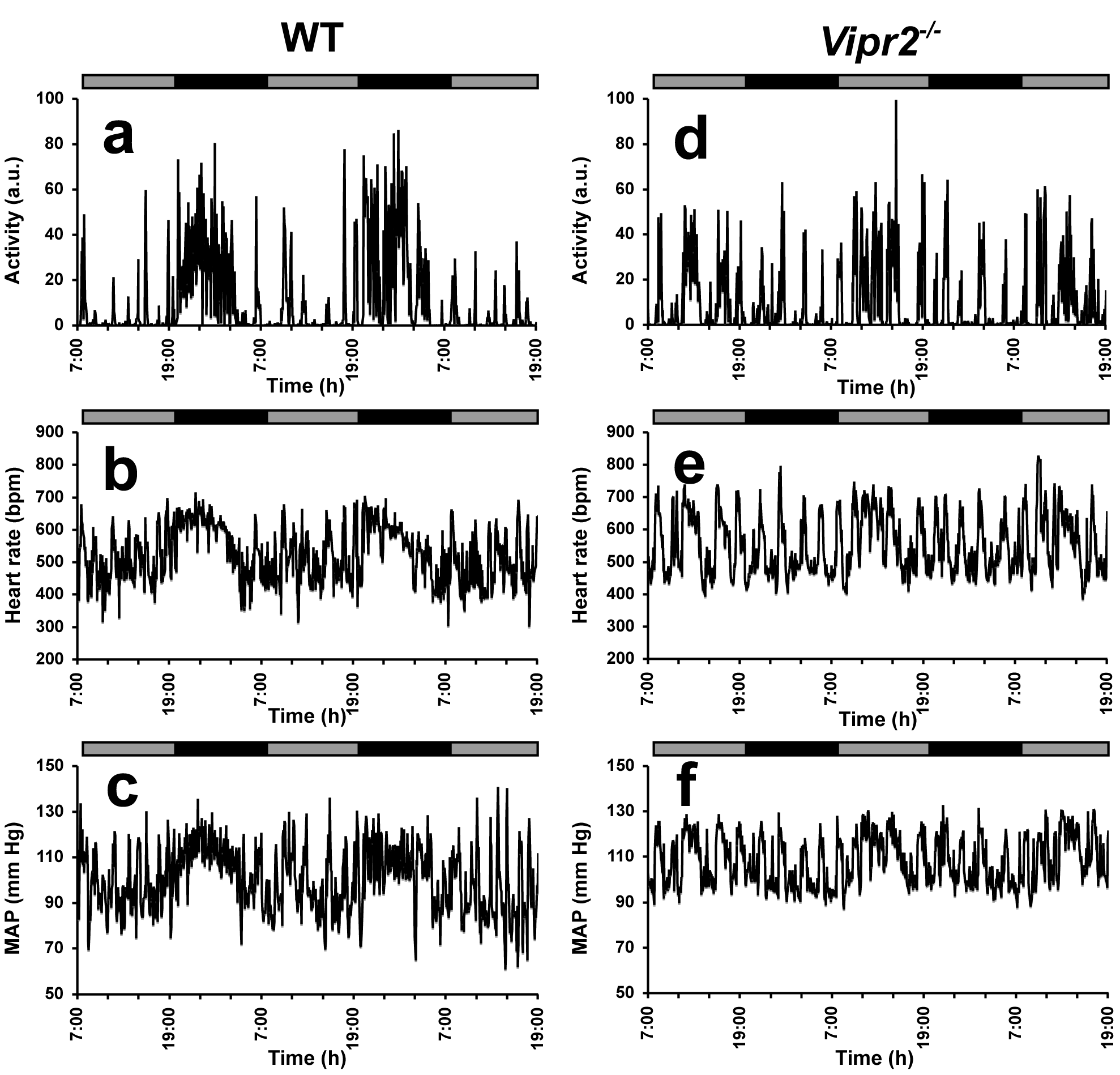

Supplement: Figure S1 — Activity (a, d), HR (b, e) and MAP (c, f) in representative individual WT (a–c) and Vipr2−/− (d–f) mice in DD conditions. The bars at the top of each panel indicate the subjective night in gray and the subjective night in black. (3.80 MB TIF) [file pone.0009783.s001.tif]

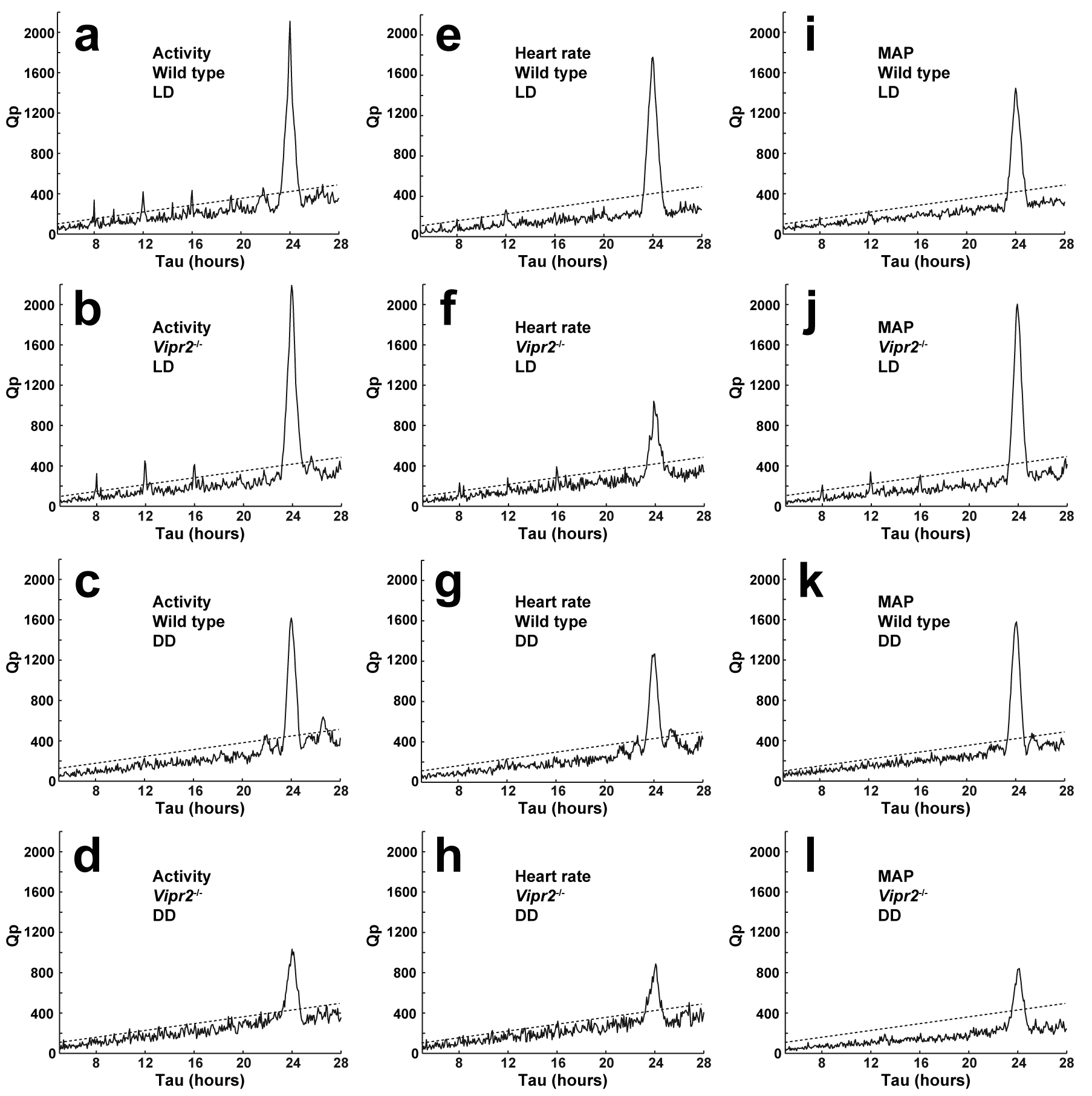

Supplement: Figure S2 — Periodogram analysis of activity, HR and MAP. Chi-square periodograms of activity (a–d), HR (e–h) and BP (i–l) from WT (a, c, e, g, i, k) and Vipr2−/− (b, d, f, h, j, l) mice (n = 5 of each genotype) over 10 day periods in a light-dark cycle (LD: a, b, e, f, i, j) and in constant conditions (DD: c, d, g, h, k, l). The Qp statistic was calculated for periods between 5 and 28 h. Dashed lines indicate the value of Qp required to achieve statistical significance (P<0.01). (3.31 MB TIF) [file pone.0009783.s002.tif]
