# Supplementary material for: Circadian Control of Mouse Heart Rate and Blood Pressure by the Suprachiasmatic Nuclei: Behavioral Effects Are More Significant than Direct Outputs
Source: PLoS One. 2010 Mar 22;5(3):e9783. doi: 10.1371/journal.pone.0009783 (PMC2842429; doi:10.1371/journal.pone.0009783)
Supplement: Table S1 — Summary of basic hemodynamics and activity for wild-type and Vipr2−/−mice under entrained (LD 12∶12) conditions. * P<0.05, ** P<0.005, compared with WT (unpaired t-test) †P<0.05, ‡P<0.01, §P<0.005, ¶P<0.001 compared with corresponding value in the light period (paired t-test). (0.07 MB PDF) [file pone.0009783.s003.pdf]

**Table S1**. Summary of basic hemodynamics and activity for wild-type and *Vipr2*$^{-/-}$ mice under entrained (LD 12:12) conditions

| Period | Variable | WT | *Vipr2*$^{-/-}$ |
|---|---|---|---|
| 24h | SAP (mm Hg) | 114 ± 5 | 119 ± 3 |
| | MAP (mm Hg) | 103 ± 6 | 108 ± 3 |
| | DAP (mm Hg) | 92 ± 5 | 98 ± 3 |
| | PP (mm Hg) | 21.9 ± 2.4 | 21.5 ± 2.2 |
| | HR (beats min$^{-1}$) | 516 ± 20 | 538 ± 12 |
| | Inactive time (%) | 27 ± 3 | 31 ± 3 |
| | Mean activity (a.u.) | 10.9 ± 0.9 | 8.8 ± 0.9 |
| | Mean (activity >0) (a.u.) | 15.3 ± 1.6 | 12.7 ± 1.0 |
| Light period | SAP (mm Hg) | 110 ± 5 | 116 ± 3 |
| | MAP (mm Hg) | 99 ± 5 | 106 ± 3 |
| | DAP (mm Hg) | 88.0 ± 4 | 95 ± 4 |
| | PP (mm Hg) | 22.2 ± 2.7 | 21.3 ± 2.2 |
| | HR (beats min$^{-1}$) | 487 ± 14 | 533 ± $11^{*}$ |
| | Inactive time (%) | 40 ± 4 | 36 ± 4 |
| | Mean activity (a.u.) | 4.5 ± 0.7 | 5.6 ± 1.1 |
| | Mean (activity >0) (a.u.) | 7.2 ± 1.0 | 8.4 ± 1.3 |
| Dark period | SAP (mm Hg) | 119 ± $4^{\dagger}$ | 122 ± 3 |
| | MAP (mm Hg) | 108 ± $6^{\S}$ | 111 ± 3 |
| | DAP (mm Hg) | 98 ± $6^{\P}$ | 100 ± 3 |
| | PP (mm Hg) | 21.6 ± 2.8 | 21.6 ± 2.2 |
| | HR (beats min$^{-1}$) | 547 ± $22^{\dagger}$ | 542 ± $12^{\ddagger}$ |
| | Inactive time (%) | 14 ± $1^{\P}$ | 26 ± $3^{**}$ |
| | Mean activity (a.u.) | 17.3 ± $2.1^{\S}$ | 12.1 ± $1.2^{\S *}$ |
| | Mean (activity >0) (a.u.) | 20.5 ± 2.7 | 16.1 ± 1.2 |

$^{*}P<0.05$, $^{**}P<0.005$, compared with WT (unpaired *t*-test)
$^{\dagger}P<0.05$, $^{\ddagger}P<0.01$, $^{\S}P<0.005$, $^{\P}P<0.001$ compared with corresponding value in the light period (paired *t*-test).
