# Supplementary material for: Circadian Control of Mouse Heart Rate and Blood Pressure by the Suprachiasmatic Nuclei: Behavioral Effects Are More Significant than Direct Outputs
Source: PLoS One. 2010 Mar 22;5(3):e9783. doi: 10.1371/journal.pone.0009783 (PMC2842429; doi:10.1371/journal.pone.0009783)
Supplement: Table S2 — Relative contributions of locomotor activity and the day/night cycle to variability in HR and BP. Percentage (mean ± SEM) of the total variance in HR and BP explained by locomotor activity and the day/night cycle, calculated using the squared correlation coefficient (R 2) measure for explained variance. (0.08 MB PDF) [file pone.0009783.s004.pdf]

Table S2. Relative contributions of locomotor activity and the day/night cycle to variability in HR and BP

		WT		*Vipr2*$^{-/-}$	
		BP	HR	BP	HR
Light-dark cycle (LD)	Activity	16.7 ± 1.0	20.5 ± 1.2	30.2 ± 1.3	22.1 ± 1.2
	Day/night	15.1 ± 1.2	11.7 ± 1.4	4.6 ± 0.9	0.6 ± 0.3
Constant conditions (DD)	Activity	17.0 ± 1.1	13.5 ± 1.0	30.6 ± 1.4	21.1 ± 1.2
	Day/night	14.8 ± 1.3	7.8 ± 1.0	4.8 ± 0.8	3.4 ± 0.7

Percentage (mean ± SEM) of the total variance in HR and BP explained by locomotor activity and the day/night cycle, calculated using the squared correlation coefficient ($\boldsymbol{R}^2$) measure for explained variance.
