# Supplementary material for: Circadian Control of Mouse Heart Rate and Blood Pressure by the Suprachiasmatic Nuclei: Behavioral Effects Are More Significant than Direct Outputs
Source: PLoS One. 2010 Mar 22;5(3):e9783. doi: 10.1371/journal.pone.0009783 (PMC2842429; doi:10.1371/journal.pone.0009783)
Supplement: Table S3 — Number and average length of wake and sleep bouts greater than 30 sec. for seven Vipr2−/− and six wild-type mice under entrained (LD 12∶12) conditions.*P<0.05, †P<0.02, ‡P<0.01, §P<0.005 compared with WT. (0.08 MB PDF) [file pone.0009783.s005.pdf]

Table S3. Number and average length of wake and sleep bouts greater than 30 sec. for seven *Vipr2*$^{-/-}$ and six wild-type mice under entrained (LD 12:12) conditions.

	**Wild-type**		***Vipr2***$^{-/-}$	
	**mean**	**SEM**	**mean**	**SEM**
**Time awake (min)**				
Total per 24 h	859	19	813	13
light	279	15	376	24‡
dark	580	17	437	32*
**Time in NREM sleep (min)**				
Total per 24 h	516	16	559	11*
light	388	16	306	22†
dark	128	16	253	28§
**Time in REM sleep (min)**				
Total per 24 h	66	4	68	3
light	54	2	38	3§
dark	12	2	30	5‡
**Number of wake bouts**				
24 h	73	8	87	19
light	55	4	40	5
dark	18	4	47	15
**Number of NREM bouts**				
24 h	97	10	112	15
light	75	6	57	4*
dark	22	4	56	14
**Number of REM bouts**				
24 h	47	4	46	2
light	38	3	26	3†
dark	9	2	19	3‡
**Average wake bout length (min)**				
24 h	12.1	1.3	11.6	2.1
light	5.0	0.3	11.4	2.1†
dark	37.9	7.1	13.8	3.4‡
**Average NREM bout length (min)**				
24 h	5.8	0.5	5.7	0.6
light	5.7	0.6	5.8	0.4
dark	6.3	0.5	5.8	1.0
**Average REM bout length (min)**				
24 h	1.5	0.1	1.6	0.03
light	1.5	0.1	1.6	0.03
dark	1.6	0.1	1.6	0.1
**Number of stage shifts**				
24 h	514	31	598	67
light	402	17	325	38
dark	110	17	272	54*
**Number of arousals**				
24 h	129	5	147	17
light	102	6	90	14
dark	27	4	57	8‡

$^{*}P$<0.05, $^{†}P$<0.02, $^{‡}P$<0.01, $^{§}P$<0.005 compared with WT.
